# Deriving GWAS summary estimates for paternal smoking in UK biobank: a GWAS by subtraction

**DOI:** 10.1186/s13104-023-06438-4

**Published:** 2023-07-30

**Authors:** Benjamin Woolf, Hannah M. Sallis, Marcus R. Munafò, Dipender Gill

**Affiliations:** 1 School of Psychological Science, University of Bristol, Bristol, UK; 2 MRC Integrative Epidemiology Unit at the University of Bristol, Bristol, UK; 3 MRC Biostatistics Unit at the University of Cambridge, Cambridge, UK; 4 Centre for Academic Mental Health, Population Health Sciences, Bristol Medical School, University of Bristol, Bristol, UK; 5 Department of Epidemiology and Biostatistics, School of Public Health, Imperial College London, London, UK; 6 Chief Scientific Advisor Office, Research and Early Development, Novo Nordisk, Copenhagen, Denmark

**Keywords:** GWAS-by-subtraction, Intergenerational Mendelian randomisation, Family GWAS, Genome Wide association studies

## Abstract

**Objective:**

To use genome-wide association study (GWAS) by subtraction, a method for deriving novel GWASs from existing summary statistics, to derive genome-wide summary statistics for paternal smoking.

**Result:**

A GWAS by subtraction was implemented using a weighted linear model that defined the child-genotype paternal-phenotype association as the child-genotype child-phenotype association minus the child-genotype maternal-phenotype association. We first use the laws of inherence to derive the weighted linear model. We then implemented the linear model to create a GWAS of paternal smoking by subtracting the summary statistics from a GWAS of maternal smoking from the summary statistics of a GWAS of the index individual’s smoking. We used a Monte-Carlo simulation to validate the model and showed that this approach performed similarly in terms of bias to performing a traditional GWAS of paternal smoking. Finally, we validated the summary statistics in a Mendelian randomisation analysis by demonstrating an association of genetically predicted paternal smoking with paternal lung cancer and emphysema.

**Supplementary Information:**

The online version contains supplementary material available at 10.1186/s13104-023-06438-4.

## Introduction

Genome-wide association studies (GWAS) are a common way of estimating genotype–phenotype associations [1]. In a GWAS, the association of each variant with the phenotype is estimated in a hypothesis-free manner [2]. GWASs typically only include ‘common’ genetic variants which occur in at least 1% of the study population. One application of GWAS summary statistics is as a source of genotype–phenotype associations for two-sample Mendelian randomisation (MR) analyses [3–5]. MR is an epidemiological design which leverages the random inheritance of genetic variants to justify the assumptions of the instrumental variable framework.

The implications of parental smoking on child outcomes are of public health importance. Because half of an individual’s genotype is a random sample of half of their mother’s genotype, one should be able to find robust associations between the child’s genotype and the maternal phenotype due to the associations between the maternal genotype and maternal phenotype and the maternal genotype and offspring’s genotype. Published GWASs summary statistics of maternal smoking allow for the investigation of the effect of an individual’s parental smoking on their (the child’s) outcomes in what has been dubbed ‘proxy gene-environment MR’ [6].

Paternal smoking is also an exposure of interest. For example, studies looking at the effect of maternal smoking on offspring birth outcomes have used paternal smoking as a negative control [7]. However, there are no published GWASs of paternal smoking. GWAS by subtraction (GWAS-BS) is a recent method for deriving novel GWASs from existing summary statistics [8]. In a traditional GWAS-BS, two or more GWASs are combined using structural equation modelling. An alternative is to use a weighted linear model (WLM) [9]. A WLM is created by combining the GWAS summary statistics using an a priori linear model (e.g., y = x_1_ + 2*x_2_—3 where x_1_ and x_2_ are the single-nucleotide polymorphism (SNP) effects being combined). Because the amount of genetic overlap between parents and children is known a priori, WLM has already been used to adjust GWAS summary statistics for dynastic effects (e.g., the association between the maternal genotype and child phenotype due to the direct inherence of the genetic variants by the child rather than in utero effects) [8].

**Fig. 1:**
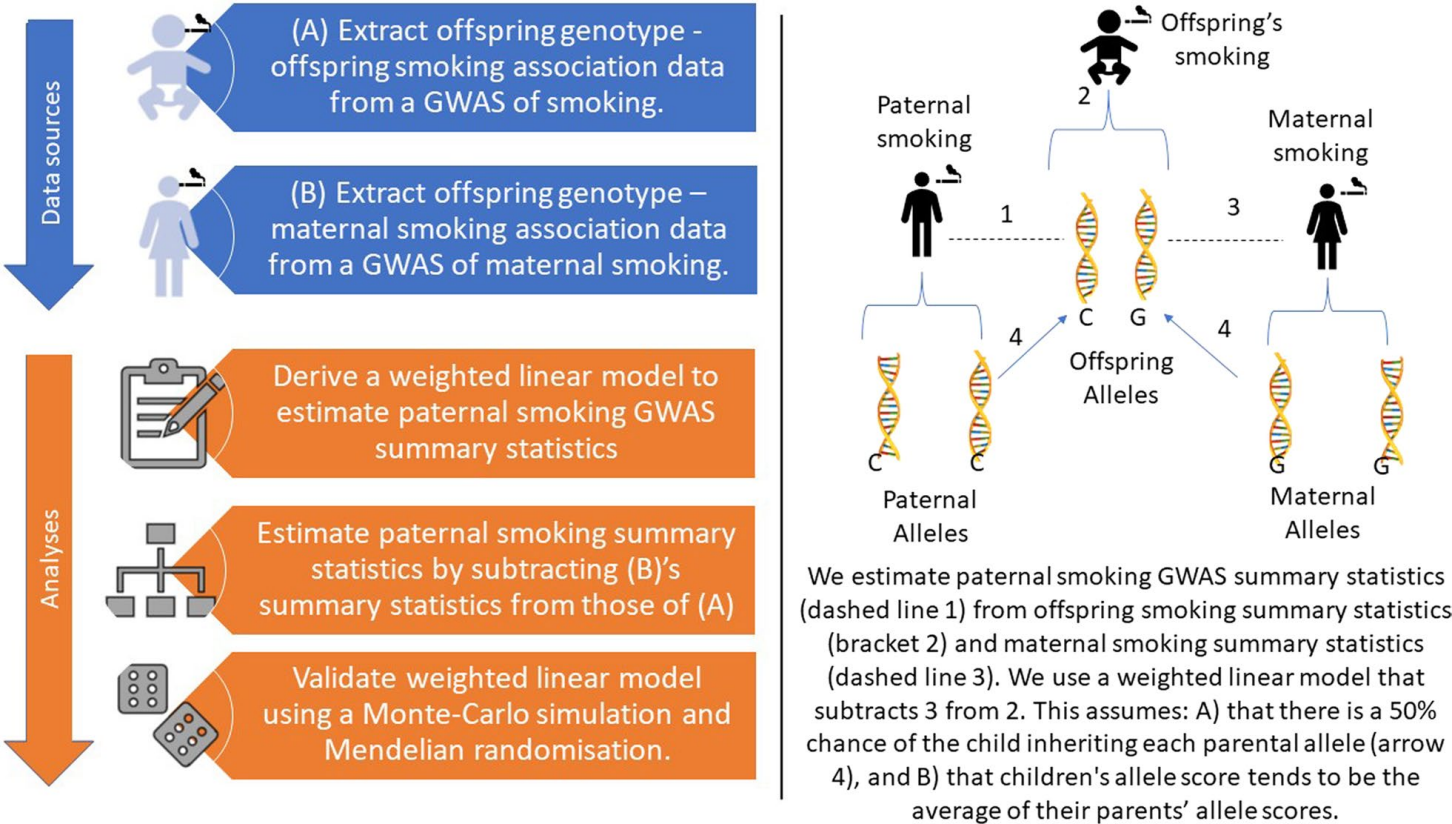
Study overview figure

Here we used a WLM and GWASs of lifetime smoking and maternal smoking to derive a GWAS of paternal smoking (Fig. 1). In brief, because the offspring’s genetic liability to smoke is half due to the inertance of the maternal genetic liability, and half due to the inherence of the paternal genetic liability, by subtracting the maternal genetic liability estimated in a GWAS from the child’s genetic liability estimated in a GWAS using a WLM we created GWAS summary statistics for paternal smoking.

## Main text

### Derivation of the linear model used in the GWAS by subtraction

An individual’s genetic risk is the average of their parent’s genetic risk. Hence:

1 $$\upbeta_{{{\rm{CG}} \_ {\text{CP}}}} = \, \frac{ {{\upbeta}_{{{\rm{FG}} \_ {\text{FP}}}} + {\upbeta}_{{{\rm{MG}} \_ {\text{MP}}}} } }{2}$$

where ${\upbeta}_{{{\text{CG}} \_ {\text{CP}}}}$ is the association between the child’s genotype and child’s phenotype (i.e. his/her genetic liability towards the phenotype), ${\upbeta}_{{{\text{FG}} \_ {\text{FP}}}}$ is the association between the father’s genotype and father’s phenotype, and ${\upbeta}_{{{\text{MG}} \_ {\text{MP}}}}$ is the association between the maternal genotype and maternal phenotype.

The association between the child’s genotype and the parental phenotype is the product of the association between the child’s genotype and the parental genotype, and the parental genotype and the parental phenotype. Therefore:

2 $${\upbeta}_{{\rm{CG}} \_ {\text{FP}}} = {\upbeta}_{{\rm{CG}} \_ {\text{FG}}} * {\upbeta}_{{\rm{FG}} \_ {\text{FP}}}$$

3 $${\upbeta}_{{\text{CG}} \_ {\text{MP}}} = {\upbeta}_{{\text{CG}} \_ {\text{MG}}} *{\upbeta}_{{\text{MG}} \_ {\text{MP}}}$$

where ${\upbeta}_{{{\text{CG}} \_ {\text{FP}}}}$ is the association between the child’s genotype and the father’s phenotype, and ${\upbeta}_{{{\text{CG}} \_ {\text{MG}}}}$ is the association between the child’s genotype and the mother's phototype.

Because the child inherits half of each parent’s genetic liability, the association between the child’s genotype and the parent’s genotype (${\upbeta}_{{{\text{CG}} \_ {\text{FG}}}}$ and ${\upbeta}_{{{\text{CG}} \_ {\text{MG}}}}$ for father’s and mother’s respectively) will be equal to 0.5. Therefore:

4 $${\upbeta}_{{{\rm{CG}} \_ {\text{FP}}}} = \, 0.5 *{\upbeta}_{{{\text{FG}} \_ {\text{FP}}}}$$

5 $${\upbeta}_{{{\text{CG}} \_ {\text{MP}}}} = \, 0.5 *{\upbeta}_{{{\rm{MG}} \_ {\text{MP}}}}$$

Therefore, by combining Eqs. (1, 4, 5) we get:

6 $${\upbeta}_{{\text{CG}} \_ {\text{FP}}} = {\upbeta}_{{\text{CG}} \_ {\text{CP}}} - {\upbeta}_{{\text{CG}} \_ {\text{MP}}}$$

Using the rules of propagation of error, the standard error of this effect would therefore be

$${se}({{{\hat{\upbeta}}_{{\rm{CG}} \_ {\rm{FP}}}}})=\sqrt{{se}({\hat{\upbeta}}_{{\rm{CG}} \_{\rm{CP}}})^{2}+{se}_({\hat{\upbeta}}_{\mathrm{CG} \_\mathrm{MP}})^{2}}$$

where ${se}({{{\hat{\upbeta}}_{{\rm{CG}} \_{\rm{CP}}}}})$ is the standard error of the estimated association between the child’s genotype and child’s phenotype, and ${se}({{{\hat{\upbeta}}_{{\rm{CG}} \_\mathrm{MP}}}})$ is the standard error of the estimated between the child’s genotype and maternal phenotype. A key of variable names in the equations can be found in Table 1.

**Table 1:** Key providing the full forms of variables used in equations

Variable initial	Manuscript section	Full form	Contribution to analyses
${\upbeta}_{{{\text{CG}} \_ {\text{CP}}}}$	Derivation of the linear model	The association between the child’s genotype and child’s phenotype (i.e. his/her genetic liability towards the phenotype)	This is estimated from a GWAS of smoking. Paternal smoking summary statistics are derived by subtracting ${\upbeta}_{{{\text{CG}} \_ {\text{MG}}}}$ from this variable
${\upbeta}_{{{\text{FG}} \_ {\text{FP}}}}$		The association between the father’s genotype and father’s phenotype	NA
${\upbeta}_{{{\text{MG}} \_ {\text{MP}}}}$		The association between the mother’s genotype and the mother’s phenotype	NA
${\upbeta}_{{{\text{CG}} \_ {\text{FP}}}}$		The association between the child’s genotype and the father’s phenotype	This is the target estimand of the WLM
${\upbeta}_{{{\text{CG}} \_ {\text{FG}}}}$		The association between the child’s genotype and the father’s genotype	NA
${\upbeta}_{{{\text{CG}} \_ {\text{MP}}}}$		The association between the child’s genotype and the mother’s phenotype	This is estimated from a GWAS of maternal smoking. Paternal smoking summary statistics are derived by subtracting this variable from ${\upbeta}_{{{\text{CG}} \_ {\text{CP}}}}$
${\upbeta}_{{{\text{CG}} \_ {\text{MG}}}}$		The association between the child’s genotype and the mother’s genotype	NA
${se}({{{\hat{\upbeta}}_{{\rm{CG}} \_{{\rm{FP}}}}}})$		Standard error of the estimate of ${\upbeta}_{{{\text{CG}} \_ {\text{FG}}}}$	Uncertainty in WLM estimates
${se}({{{\hat{\upbeta}}_{{\rm{CG}} \_{{\rm{FP}}}}}})$		Standard error of the estimate of ${\upbeta}_{{{\text{CG}} \_ {\text{CP}}}}$	Uncertainty in the estimates from the smoking GWAS
${se}({{{\hat{\upbeta}}_{{\rm{CG}} \_{{\rm{FP}}}}}})$		Standard error of the estimate of ${\upbeta}_{{{\text{CG}} \_ {\text{MP}}}}$	Uncertainty in the estimates from the maternal smoking GWAS
F_i_	Validation situation	Allele inherited from the father	NA
M_i_		Allele inherited from the mother	NA
C_g_		Child’s unweighted allele score	Exposure in all GWASs
W		Variant-phenotype association (i.e. allele score weights)	NA
C_p_		Child’s phenotype	Outcome in GWAS of smoking
M_g_		Maternal allele score (i.e. with both inherited and non-inherited allele)	NA
M_p_		Mother's phenotype	Outcome in GWAS of maternal smoking
F_p_		Father’s phenotype	Outcome in the traditional GWAS of paternal smoking

*WLM* weighted linear model, *GWAS* Genome-Wide Association Study

### Validation of the linear model

To validate this linear model, we ran a simulation. We report our simulations using the ADEMP (aims, data-generating mechanisms, estimands, methods, and performance measures) approach [10]:

#### Aims

To validate the proposed WLM as a method for producing unbiased estimates of the association between the child’s genotype and the father’s phenotype.

#### Data-generating mechanisms

We simulated both the inherited maternal and paternal genotypes as two independent but identically distributed one-level binomial distributions

$${\text{F}}_{{\text{i}}} \sim {\text{B}}(0.5, 1)$$

$${\text{M}}_{{\text{i}}} \sim {\text{B}}(0.5, 1)$$

For the paternal and maternal inherited genetic variants respectively.

The child’s genotype was then defined as

$${\text{C}}_{{\text{g}}} = {\text{ F}}_{{\text{i}}} + {\text{ M}}_{{\text{i}}}$$

The genotype–phenotype association was defined as

$${\text{W }}\sim {\text{ N}}(1, 0.006)$$

The child’s phenotype was then the product of both the parental variants and a random normal error:

$${\text{C}}_{{\text{p}}} = {\text{ C}}_{{\text{g}}} *{\text{W }} + {\text{ N}}\left({0, 1} \right)$$

The maternal genotype was defined as

$${\text{M}}_{{\text{g}}} = {\text{ W}}( {0.5, 1} ) \, + {\text{ M}}_{{\text{i}}}$$

The maternal and paternal phenotypes were then respectively defined as:

$${\text{M}}_{{\text{p}}} = {\text{ M}}_{{\text{g}}} *{\text{W }} + {\text{ N}}\left( {0,{1}} \right)$$

$${\text{F}}_{\rm{p}}=\left({{\text{F}}_{{\rm{i}}}+{\text{B}}\left({0.5,{1}}\right)}\right)*{\text{W}}+{\text{N}}\left({0,{1}}\right)$$

#### Estimand

The association between the child’s genotype and the paternal phenotype.

#### Methods

We compare two methods for estimating the association between the child’s genotype with the child’s phenotype and the maternal phenotype by (1) regressing the paternal phenotype on the child’s genotype. This method would produce results analogous to those of a traditional GWAS of paternal smoking. (2) Regressing the maternal phenotype on the child’s genotype and the child’s phenotype on the child’s genotype and combining these using the proposed WLM.

#### Performance measure

We then calculated the mean bias and Monte-Carlo standard error in the two estimates.

#### Additional simulations

To further validate the model for a wider range of settings we additionally ran the simulation using the beta and minor allele frequency values for the 126 genome wide significant SNPs from the Wootton et al. GWAS of smoking as the beta (i.e. W above) minor allele frequency values (i.e. probabilities for F_i_, M_i_, F_p_, and M_g_ above) for the simulation. We additionally ran the simulation using the minimum, 1st quartile, mean, median, 3rd quartile, and maximum values of the above two parameters from the GWAS (Additional file 1: Table S1).

#### Results of the simulation

The mean bias by directly regressing the paternal phenotype on the child’s genotype was 0.001 (95% CI 0.003 to − 0.001), while the mean bias in the WLM was 0.000 (95% CI 0.002 to − 0.002). This implies that our WLM should perform similarly, in terms of bias, to a GWAS of paternal smoking.This conclusion was reinforced by the additional simulations (Additional file 1: Table S1).

### Creation and validation of GWAS summary statistics

We implemented the above WLM using the GWAS of lifetime smoking by Wootton and colleagues and the GWAS of maternal smoking during pregnancy by Elsworth and colleagues [11, 12]. Both GWAS were created using the Medical Research Council Integrated Epidemiology Unit (MRC IEU) GWAS pipeline, which is described in detail elsewhere [13]. To have comparable units in both GWASs we converted both GWAS to have units on the standardised mean difference scale. We standardised the lifetime smoking summary statistics by dividing the beta and standard error of the summary statistics by the standard deviation of lifetime smoking (0.6940093). We standardised the maternal smoking GWAS summary statistics by first converting them into log odds ratios by dividing the effect estimate and standard error by (121634/397732)*(1-(121634/397732)) (i.e., p[1-p], where p is the prevalence of maternal smoking during pregnancy), and then convert the log odds into a standardised mean difference by dividing them by ${(}\pi *{3}^{-0.5})$ [13, 14] (Additional file 2: Fig S1).

**Fig. 2:**
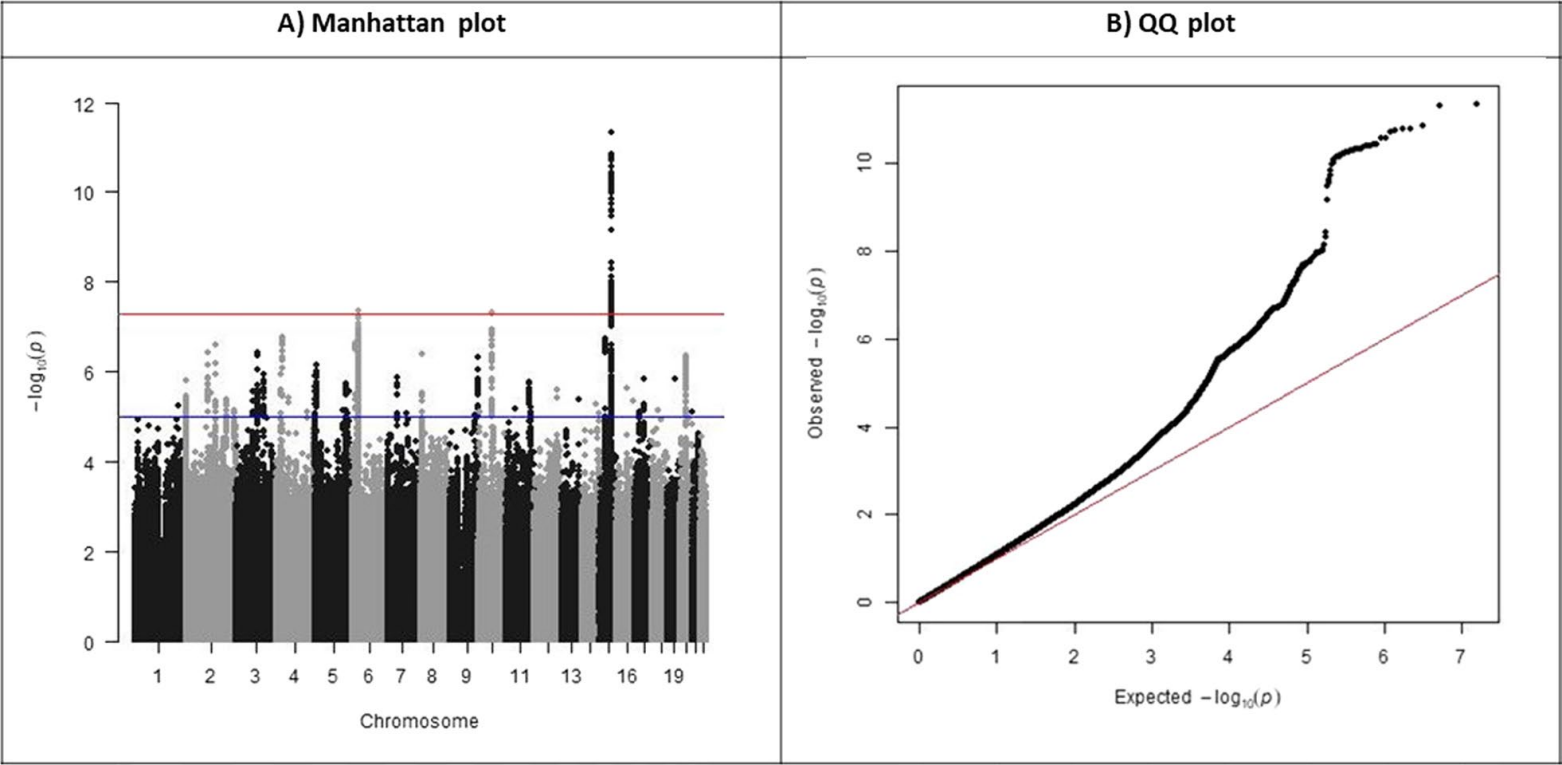
Manhattan and QQ plots for the GWAS of paternal smoking

The resulting GWAS had a genomic control inflation factor (λ_GC_) of 1.091 (SE = 0.027). Figure 2 presents the Manhattan and QQ plot for the GWAS. The λ_GC_ and QQ plot imply that the test statistics are larger than what would be expected by chance, and therefore the potential presence of residual bias in our summary statistics. The reduction in the number of hits in the Manhattan plot when compared to the maternal smoking and lifetime smoking GWAS implies that the WLM has reduced power compared to these GWASs. Both the GWASs used the UK Biobank (UKB), and had adjusted for the UKB genotyping chip. Following general advice, we additionally created a secondary GWAS of paternal smoking from the same GWASs, but without adjusting for genotyping chip. However, not adjusting for genotyping chip appears to result in more biased estimates (see the Additional file for more details).

### Validation of the GWAS summary statistics

We validated the GWAS summary data by testing using MR that variants predicting paternal smoking associated with paternal lung cancer and emphysema/bronchitis. In brief, we selected SNPs with a 5 × 10^–6^ association in our paternal smoking GWAS. The p-value threshold was chosen to boost the number of SNPs included in the analysis, and therefore power while ensuring reasonably strong instrument strength. As a sensitivity analysis, we also selected SNPs at 5 × 10^–7^ and 5 × 10^–8^ p-value thresholds. We clumped with an r^2^ of 0.001 and kb of 10,000. We additionally implemented the False Discovery Rate Inverse Quantile Transformation Winner’s curse correction on the exposure GWASs to correct for the effect of Winner’s curse [15]. We then used the Elsworth and colleagues UKB GWAS in the MRC IEU OpenGWAS platform as a source of paternal outcome data [11]. Details on genotyping, quality control, and phenotyping can be found in the original publications and on the UKB website (https://biobank.ndph.ox.ac.uk/ukb/search.cgi).

We implemented the MR analysis using the TwoSampleMR R package [16, 17]. We harmonised the data, and allowed TwoSampleMR to removed palindromic SNPs whose effect allele could not be inferred using based on minor allele frequency. SNP effects were combined using the inverse-variance weighted (IVW) meta-analysis with multiplicative random effects.

Our paternal GWAS had relatively strong instruments (mean F = 24 from 26 SNPs). As expected, in our primary analysis we found positive associations between each standard deviation of genetically proxied paternal smoking and the log odds of paternal lung cancer (risk difference per standard deviation (SD) increase in smoking = 0.754 (se = 0.362, p = 0.034) and emphysema/bronchitis (risk difference per SD increase in smoking = 1.014 (se = 0.285, p = 0.0004). Our secondary analyses using more stringent p-value thresholds to select SNPs found results in the same direction, but larger beta values. Using a 5 × 10^–7^ p-value threshold (N SNPs = 12, F = 27) the log odds of paternal lung cancer (risk difference per SD increase in smoking = 1.050 (se = 0.726, p = 0.148) and emphysema/bronchitis (risk difference per SD increase in smoking = 1.411 (se = 0.510, p = 0.006). Using a 5 × 10^–7^ p-value threshold (N SNPs = 3, F = 35) the log odds of paternal lung cancer (risk difference per SD increase in smoking = 1.452 (se = 2.774, p = 0.601) and emphysema/bronchitis (risk difference per SD increase in smoking = 3.132 (se = 0.943, p = 0.0009). The lack of a significant effect for lung cancer with the more stringent p-values probably reflects the reduction of power from using fewer SNPs, and that there are around 20% fewer lung cancer cases than emphysema/bronchitis cases (37,443 vs 46,263).

## Limitations

To the best of our knowledge, this is the first application of WLMs to derive GWAS summary statistics for one parent when observations have only been made on the offspring and another parent.

Both our WLM and a direct GWAS of paternal smoking could be affected by assortative mating. Specifically, we would expect a direct GWAS of parental smoking to be inflated by assortative mating because the other parent’s genotype would confound the association with the offspring’s genotype. The WLM will then also be biased to the extent that the inflation of the SNP effects in the GWAS of parental smoking is not proportional to the inflation of the GWAS of lifetime smoking. In addition, our WLM may also be biased by residual population structure in either the lifetime smoking or maternal smoking GWASs, or indirect genetic effects in the lifetime smoking GWAS [18].

We created this GWAS primarily for use within a Mendelian randomisation design. Multivariable MR (MVMR) is an extension of MR to include multiple exposures. Estimation of the (conditional) instrument strength for an MVMR analysis requires knowing the correlation between the exposures, or assuming that the correlation is zero. The latter is less conservative, however, because we lack the phenotype exposure data the prior cannot be estimated. An alternative may be to use the correlations of maternal smoking with the relevant phenotypes as a proxy. Doing so should produce more conservative estimates of the instruments than assuming no correlation. The correlation between maternal and paternal smoking can, however, be estimated from the existing literature on assortative mating [19].

Finally, a limitation of linear models is that they can result in under-fitting of data, e.g. due to non-differential measurement error. We are, however, not aware of other external GWAS or biobank data form which we could further validate our GWAS by comparing our results too.

In this research note we have described and validated the creation of a GWAS of paternal smoking via a GWAS-by-subtraction. We hope that they will further facilitate the study of intergenerational effects.

## Supplementary Information

**Additional file 1.** Table S1. Results of the additional simulations.

**Additional file 2.** Details of the paternal GWAS not adjusted for UK Biobank genotyping chip. Figure S1. Manhattan and QQ plots for the two paternal smoking GWAS.

## Data Availability

The code and GWAS summary statistics used in this study is available from https://doi.org/10.17605/OSF.IO/SPKMT.

## References

[1] Benn M, Nordestgaard BG. From genome-wide association studies to mendelian randomization: novel opportunities for understanding cardiovascular disease causality, pathogenesis, prevention, and treatment. Cardiovasc Res. 2018. 10.1093/cvr/cvy045/487705. 29471399 10.1093/cvr/cvy045

[2] Mitchell R, Hemani G, Dudding T, Corbin L, Harrison S, Paternoster L. UK Biobank Genetic Data: MRC-IEU Quality Control, version 2. https://research-information.bris.ac.uk/en/datasets/uk-biobank-genetic-data-mrc-ieu-quality-control-version-2

[3] Woolf B, Di Cara N, Moreno-Stokoe C, Skrivankova V, Drax K, Higgins JPT, et al. Investigating the transparency of reporting in two-sample summary data Mendelian randomization studies using the MR-base platform. Int J Epidemiol. 2022;46(6):815. 10.1093/ije/dyac074 PMC9749715 35383846

[4] Bowden J, Del Greco MF, Minelli C, Davey Smith G, Sheehan NA, Thompson JR. Assessing the suitability of summary data for two-sample Mendelian randomization analyses using MR-Egger regression: the role of the I2 statistic. Int J Epidemiol. 2016;45(6):1961–74. 27616674 10.1093/ije/dyw220 PMC5446088

[5] Burgess S, Foley CN, Zuber V. Inferring causal relationships between risk factors and outcomes from genome-wide association study data. Annu Rev Genomics Hum Genet. 2018;19(1):303–27. 29709202 10.1146/annurev-genom-083117-021731 PMC6481551

[6] Yang Q, Millard LAC, Davey SG. Proxy gene-by-environment Mendelian randomization study confirms a causal effect of maternal smoking on offspring birthweight, but little evidence of long-term influences on offspring health. Int J Epidemiol. 2020;49(4):1207–18. 31834381 10.1093/ije/dyz250 PMC7660158

[7] Taylor AE, Carslake D, de Mola CL, Rydell M, Nilsen TIL, Bjørngaard JH, et al. Maternal smoking in pregnancy and offspring depression: a cross cohort and negative control study. Sci Rep. 2017;7(1):12579. 28974730 10.1038/s41598-017-11836-3 PMC5626710

[8] Investigating the genetic architecture of noncognitive skills using GWAS-by-subtraction | Nature Genetics. https://www.nature.com/articles/s41588-020-00754-2 10.1038/s41588-020-00754-2 PMC7116735 33414549

[9] Maternal and fetal genetic effects on birth weight and their relevance to cardio-metabolic risk factors | Nature Genetics. https://www.nature.com/articles/s41588-019-0403-1 10.1038/s41588-019-0403-1 PMC6522365 31043758

[10] Morris TP, White IR, Crowther MJ. Using simulation studies to evaluate statistical methods. Stat Med. 2019;38(11):2074–102. 30652356 10.1002/sim.8086 PMC6492164

[11] Elsworth B, Lyon M, Alexander T, Liu Y, Matthews P, Hallett J, et al. The MRC IEU OpenGWAS data infrastructure. bioRxiv. 2020. 10.1101/2020.08.10.244293v1.

[12] Wootton RE, Richmond RC, Stuijfzand BG, Lawn RB, Sallis HM, Taylor GMJ, et al. Evidence for causal effects of lifetime smoking on risk for depression and schizophrenia: a Mendelian randomisation study. Psychol Med. 2020;50(14):2435–43. 31689377 10.1017/S0033291719002678 PMC7610182

[13] Ruth Mitchell E. MRC IEU UK Biobank GWAS pipeline version 2. data.bris. 2019. https://data.bris.ac.uk/data/dataset/pnoat8cxo0u52p6ynfaekeigi

[14] Higgins J, Thomas J, Chandler J, Cumpston M, Li T, Page M, et al. Cochrane Handbook for Systematic Reviews of Interventions version 6.3. Cochrane; 2022. www.training.cochrane.org/handbook.

[15] Bigdeli TB, Lee D, Riley BP, Vladimirov V, Fanous AH, Kendler KS, et al. FIQT: a simple, powerful method to accurately estimate effect sizes in genome scans. bioRxiv. 2015. 10.1101/019299v1.

[16] R Core Team. R: A language and environment for statistical computing. R Foundation for Statistical Computin. 2021. https://www.R-project.org/

[17] Hemani G, Zheng J, Elsworth B, Wade KH, Haberland V, Baird D, et al. The MR-base platform supports systematic causal inference across the human phenome. Elife. 2018;7:e34408. 29846171 10.7554/eLife.34408 PMC5976434

[18] Kong A, Thorleifsson G, Frigge ML, Vilhjalmsson BJ, Young AI, Thorgeirsson TE, et al. The nature of nurture: effects of parental genotypes. Science. 2018;359(6374):424–8. 29371463 10.1126/science.aan6877

[19] Langley K, Heron J, Davey Smith G, Thapar A. Maternal and paternal smoking during pregnancy and risk of ADHD symptoms in offspring: testing for intrauterine effects. Am J Epidemiol. 2012;176(3):261–8. 22791738 10.1093/aje/kwr510 PMC3406617

