# Correction: Deriving GWAS summary estimates for paternal smoking in UK biobank: a GWAS by subtraction

**DOI:** 10.1186/s13104-024-06972-9

**Published:** 2025-02-17

**Authors:** Benjamin Woolf, Hannah M. Sallis, Marcus R. Munafò, Dipender Gill

**Affiliations:** 1 School of Psychological Science, University of Bristol, Bristol, UK; 2 MRC Integrative Epidemiology Unit at the University of Bristol, Bristol, UK; 3 MRC Biostatistics Unit at the University of Cambridge, Cambridge, UK; 4 Centre for Academic Mental Health, Population Health Sciences, Bristol Medical School, University of Bristol, Bristol, UK; 5 Department of Epidemiology and Biostatistics, School of Public Health, Imperial College London, London, UK; 6 Chief Scientific Advisor Office, Research and Early Development, Novo Nordisk, Copenhagen, Denmark

**Correction: BMC Research Notes (2023) 16:159**

10.1186/s13104-023-06438-4

Following the publication of the original article [1], the authors realised that their choice of notation for the equations used is, at points, unclear and may be misinterpreted by the reader.

Across all instances in the manuscript, they would like to change the letter ‘B’ in their notation to the letter ‘β’, and also change ‘B’ in the equation defining their simulation setup to ‘W’. In addition, they would like to correct a typo and replace the term ‘father’ with ‘mother’.
