# Supplementary material for: Deriving GWAS summary estimates for paternal smoking in UK biobank: a GWAS by subtraction
Source: BMC Res Notes. 2023 Jul 30;16:159. doi: 10.1186/s13104-023-06438-4 (PMC10387204; doi:10.1186/s13104-023-06438-4)
Supplement: Supplementary file 2 — Additional file 2. Details of the paternal GWAS not adjusted for UK Biobank genotyping chip. Figure S1. Manhattan and QQ plots for the two paternal smoking GWAS. [file 13104_2023_6438_MOESM2_ESM.docx]

**Supplement**

Both genome-wide association studies (GWASs) used the UK Biobank (UKB), and had adjusted for the UKB genotyping chip. Following general advice, we additionally created a secondary GWAS of paternal smoking from the same GWASs, but without adjusting for genotyping chip. The chip adjusted GWAS had a genomic control inflation factor (λ_GC_) of 1.091 (standard error, SE = 0.027), while the non-chip adjusted GWAS had an inflation factor of 1.066 (SE = 0.024). Supplementary Figure S1 presents the Manhattan and QQ plots for the two GWASs. The latter implies slightly greater inflation in the non-chip adjusted GWAS.

In the applied validation, our paternal GWAS had relatively strong instruments (mean F = 24 from 26 and 31 single-nucleotide polymorphisms for the chip adjusted GWAS and no chip adjustment GWAS respectively). As expected, we found positive associations between each standard deviation (SD) of genetically proxied paternal smoking and the log odds of paternal lung cancer (risk difference per SD increase in smoking = 0.754 (SE = 0.362, p = 0.034) for the chip adjusted analysis and 0.348 (0.204, p = 0.088) for the no chip adjustment analysis) and emphysema/bronchitis (risk difference per SD increase in genetically paternal smoking = 1.014 (se = 0.285, p < 0.001) for the chip adjusted analysis and 0.062 (se = 0.020, p < 0.002) for the no chip adjustment analysis). Since the non-chip adjusted estimates are greatly deflated, this represent the effect of confounding by genotype chip inflating the variant-exposure association more than the variant-outcome association (1,2).

This applied validation implies that caution is required when applying the non-chip adjusted GWAS summary statistics which we created here. It is suggested that UKB GWAS adjust for genotyping chip in case there is a confounding effect due to the differential sampling of people for the UK Believe study based on their lung function. Our interpretation of the inflation of the MR estimates in the no-chip analysis is that our paternal GWAS is confounded by genotyping chip when this is not adjusted for.

Refences

1. Mitchell R, Hemani G, Dudding T, Corbin L, Harrison S, Paternoster L. UK Biobank Genetic Data: MRC-IEU Quality Control, version 2 [Internet]. [cited 2022 Jun 22]. Available from: https://research-information.bris.ac.uk/en/datasets/uk-biobank-genetic-data-mrc-ieu-quality-control-version-2

2. Woolf B, Gill D, Sallis H, Munafo M. The UK BiLEVE and Mendelian randomisation: Using multivariable instrumental variables to address “damned if you, dammed if you don’t” adjustment problems. [Internet]. medRxiv; 2022 [cited 2022 Oct 28]. p. 2022.10.25.22281084. Available from: https://www.medrxiv.org/content/10.1101/2022.10.25.22281084v1

**Figure S1: Manhattan and QQ plots for the two paternal smoking GWAS.**

|  | **Chip adjusted** | **Non-chip adjusted** |
| --- | --- | --- |
| **Manhattan plot** | 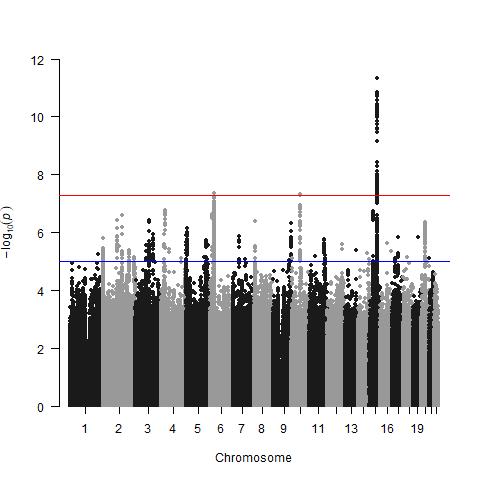 | 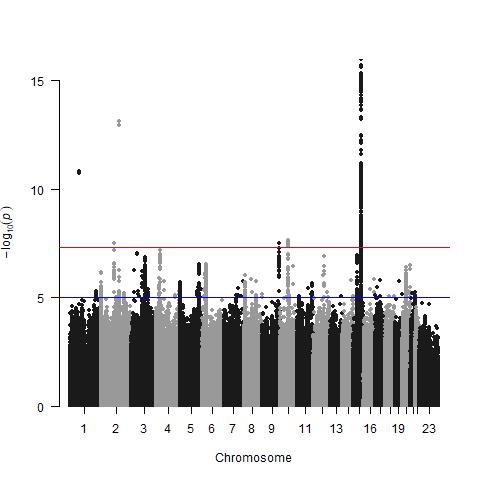 |
| **QQ plot** | 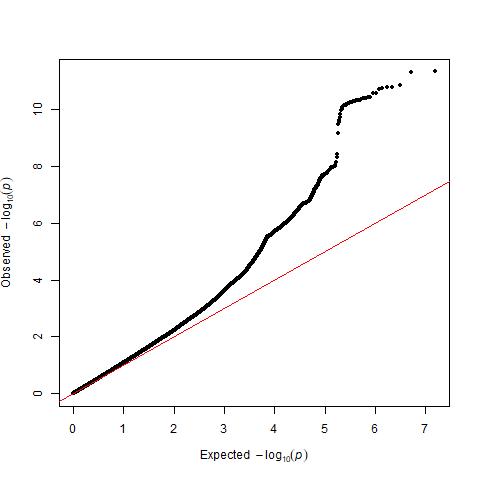 | 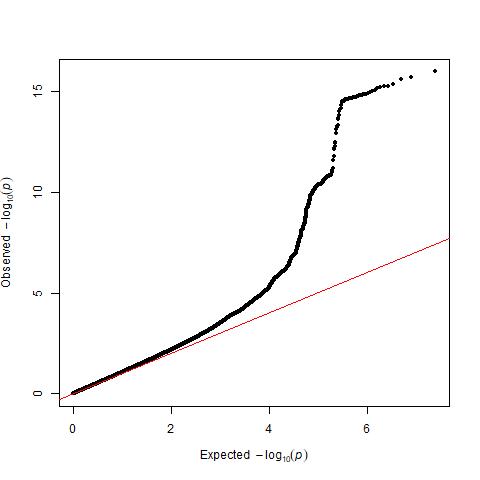 |
